# Eye Gaze Entropy Reflects Individual Experience in the Context of Driving

**DOI:** 10.3390/e28010008

**Published:** 2025-12-20

**Authors:** Karina Arutyunova, Evgenii Burashnikov, Nikita Timakin, Ivan Shishalov, Andrei Filimonov, Anastasiia Bakhchina

**Affiliations:** Cognitive Systems Lab, Harman Research, Harman International, 30001 Cabot Dr, Novi, MI 48377, USA; evgeny.burashnikov@harman.com (E.B.); nikita.timakin@harman.com (N.T.); anastasiya.bakhchina@harman.com (A.B.)

**Keywords:** eye gaze, eye tracking, entropy, individual experience, driving experience, driving

## Abstract

Eye gaze plays an essential role in the organisation of human goal-directed behaviour. Stationary gaze entropy and gaze transition entropy are two informative measures of visual scanning in different tasks. In this work, we discuss the benefits of these eye gaze entropy measures in the context of driving behaviour. In our large-scale study, participants performed driving tasks in a simulator (N = 380, 44% female, age: 20–73 years old) and in on-road urban environments (N = 241, 44% female, age: 19–74 years old). We analysed measures of eye gaze entropy in relation to driving experience and compared their dynamics between the simulator and on-road driving. The results demonstrate that, in both driving conditions, gaze transition entropy is higher, whereas stationary gaze entropy is lower, in more experienced drivers of both genders. This suggests that gaining driving experience may be accompanied by a decrease in overall gaze dispersion and an increased unpredictability of visual scanning behaviour. These results are in line with previously reported trends on experience-related dynamics of eye gaze entropy measures. We discuss our findings in the framework of the system-evolutionary theory, which explains the organisation of behaviour through the history of individual development, corresponding to the growing complexity of individual–environment interactions. Experience-related dynamics of eye gaze complexity can be a useful factor in the development of practical applications, such as driver monitoring systems and other human–machine interfaces.

## 1. Introduction

Vision is an active and predictive process [1] that provides the most comprehensive way for achieving adaptive outcomes within interactions with the environment for humans [2,3,4]. Eye movement activity is an integral part of behaviour and depends on characteristics of the visual scene, but is also impacted by individual experience [5], motivation [6,7], psychophysiological states, including increased cognitive load [8,9], fatigue [10], and emotion [11], among other factors [1]. Eye movement in tasks involving naturalistic viewing is normally described using spatial and temporal gaze parameters. Saccades and fixations on regions of the visual field within the environment are directly involved in the organisation of current behaviour. Saccades, i.e., fast ballistic eye movements that bring regions of interest in the environment onto the fovea (the most sensitive part of the retina), are critical for navigating the visual world. Fixations refer to moments when a person’s eyes focus on a particular point in the visual environment with which an individual is engaged.

Eye gaze entropy describes eye movement behaviour by quantifying the spatial distribution (stationary gaze entropy, SGE) and transition patterns (gaze transition entropy, GTE) of eye movements [12]. SGE measures the overall uncertainty in where a person looks during a given viewing period. GTE quantifies the uncertainty associated with the next gaze location relative to the current position of the eyes.

Shiferaw and colleagues [13] propose to view eye movements and gaze control as a complex system of spatial prediction, and SGE and GTE are considered within the model of gaze orientation. The authors describe how Shannon’s equation of entropy and conditional entropy can be applied to gaze analysis and propose a model of entropy to measure gaze control complexity. They propose to view GTE as an estimate of visual scanning efficiency that underlies overall gaze dispersion measured by SGE.

It is also hypothesised that GTE can be used for estimating the level of top-down modulation in gaze control, suggesting that there is an optimal range of GTE for a given task, which corresponds to the level of uncertainty relative to the complexity of the visual environment and task demands [14,15]. An increase in GTE may indicate stronger top-down modulation, but if it is increased above optimum (e.g., in the case of anxiety), it may provide interference to gaze behaviour. Decreased GTE, consequently, may be associated with reduced top-down modulation and may negatively affect performance. Depending on task-specific visual requirements, non-optimal shifts in GTE can lead to an increase or reduction in SGE, describing overall spatial dispersion of gaze, and such dynamics may reflect a mismatch between task requirements and gaze allocation.

Eye gaze entropy, in general, depends on the task and its complexity. For example, it has been shown that gaze entropy reflects surgical task load, with higher SGE observed in more complex tasks [16]. In the aviation context, gaze entropy was also sensitive to task load [17,18]. However, in fighter pilots, SGE was shown to decrease in emergency situations considered as more complex tasks, compared to non-emergency situations [17]. Although it may be argued that emergency situations also trigger a stress-like response, which is known to decrease the complexity of different entropy measures in physiology [19,20]. In another study, increased task difficulty was associated with reductions in both SGE and GTE measures in low-time pilots [18]. In a follow-up study, it was found that pilots’ gaze entropy measures varied across different stages of flight, and when compared within such stages, more difficult tasks were accompanied by higher gaze complexity measures, and this was the case for both SGE and GTE [21]. The authors note that dynamic gaze analyses can be used as robust measures of task difficulty and pilot flight hours. Thus, gaze behaviour constantly adjusts to meet specific task demands, whether it is required to extract more complex information or focus attention on very specific parts of the visual scene.

Studies show that eye movement patterns often differ based on individual experience. When comparing eye gaze dynamics between experts and novices in different tasks, research shows that novices make significantly more fixations than experts, whose eye movements tend to be longer and with larger saccade amplitudes, leaving larger areas of the visual scene unattended [22]. Kosel and colleagues [23] found that expert teachers’ scan paths were more complex and included more frequent revisits of all students, and that experts transferred their attention between all students with equal probability, overall demonstrating higher gaze entropy values. Moreover, scan path patterns were more similar within the expert group, compared to novice teachers. Qualitative scan path analysis indicated that experts’ scan paths are more guided by strategy, while novice teachers’ scan paths were more distracted by salient events irrelevant to current tasks. In contrast, gaze patterns of expert operators of bilateral teleoperation systems were shown to be more focused and goal-oriented gaze compared to less structured and more erratic behaviour of novices, and the experts had lower values of SGE and GTE [24].

Multiscale entropy analysis has been used to evaluate the dynamics of complexity of eye movements in novice participants gaining experience and completing various tasks in virtual reality environments, including smooth pursuit, vergence, reading, and video viewing [25]. The results showed that as viewing time and time on task extended, along with participants’ experience of interaction with the virtual environment, the complexity of gaze patterns consistently increased.

Ocular activity is highly relevant to driving behaviour, which requires constant engagement with a dynamic visual scene. Gaze entropy measures are shown to be useful in differentiating between age groups and combinations of tasks during driving [9]. SGE was shown to be the best metric for distinguishing between baseline driving and driving with increased visual-spatial task load. SGE was decreasing with increased task demand. Compared to distributional measures of eye movement behaviour, such as fixation dwell time and mean saccade amplitude, SGE was better at distinguishing between younger and older drivers; it was also better at discriminating between conditions of driving with different visual-spatial load. Chung and colleagues [26] explored eye gaze entropy and other metrics in drivers with different experience. They found that more experienced drivers had higher SGE and GTE values, compared to novice drivers. Novice drivers were also shown to have longer dwell time on driving relevant areas of interest and longer fixations on the navigation system and dashboard than the experienced group, who performed more gaze transitions between side mirrors and surrounding traffic conditions.

Entropy gaze measures are known to reflect the driver’s state. Shiferaw et al. [27] reported an increase in SGE and GTE during driving following one night of total sleep deprivation, which predicted deterioration in driving performance. Drivers under the influence of alcohol demonstrate a reduction in GTE accompanied by an increased SGE, which suggests more scattered viewing; moreover, such changes in eye movement mediated the impact of alcohol intoxication on measures of driving performance [15]. In other words, reduced GTE and increased SGE indicated a less structured spatial gaze dispersion.

In a recent study, Zhang and colleagues [28] utilised the transfer entropy approach for quantifying eye–head coordination during driving and demonstrated that eye movements are most frequently preceded by head movements in the context of driving in a virtual environment; they also observed some examples of the influence of eye–head coordination on driving behaviour and performance. SGE and GTE were used in studies aimed at improving road safety, such as manipulating tunnel delineators and measuring gaze complexity in different conditions [29].

In this work, we consider ocular activity within the system-evolutionary theory [30,31], which is based on the concept of functional systems comprised by diverse body and brain components [32]. Their cooperative activity leads to adaptive results in the organism-environment relations. As an example, it is highlighted that breathing and blood flow during similar actions can be very different, as their characteristics depend on achieved behavioural outcomes. The system-evolutionary theory expands this idea by viewing neurons and cells as “organisms within the organism”, where each cell’s activity satisfies its metabolic needs through interaction with the environment and other cells [31]. This collective activity, resulting in a new relationship between the organism and the environment, comprises a new functional system with active cells during the adaptive process. The actualization of such systems supports the realisation of eye gaze movements considered as behavioural acts contributing to subjective experience. From this perspective, vision is an active process of the organism–environment interactions, not just passive processing of external visual information. Thus, a functional system is a dynamic organisation of component activities across various body and brain regions, ensuring adaptive success for the organism. Within this framework, eye gaze entropy emerges from the cooperation of the eyes’ activity with other components of functional systems, including neuronal groups. This approach incorporates some characteristics of the embodied cognition concepts [33] and implies that eye gaze entropy varies with the behavioural outcomes achieved during organism–environment interactions. In this study, we aim to explore the dependency of eye gaze entropy on the structure of individual experience in the context of driving behaviour.

Thus, based on the above, we consider SGE and GTE as potential system-level descriptors of how gaze is distributed and sequenced within the visual field during driving. SGE captures the spatial dispersion of gaze points over time, while GTE quantifies the unpredictability of transitions between gaze locations. These measures do not, in themselves, specify which objects or regions are being fixated (e.g., lane markings, mirrors, or hazards), but instead index the structure of sampling behaviour given a particular visual environment during driving. Based on the theoretical approach and prior research discussed above, we hypothesize that experience and practice in solving specific tasks in the interactions with the visual environment are accompanied by less stereotyped and predictable ocular activity, which is reflected in more complex but structured patterns of eye movements. Our central hypothesis is that more experienced drivers develop gaze strategies that are better tuned to the task demands and structure of the environment, and that this tuning will be reflected in systematic changes in the dispersion and organisation of gaze that can be quantitatively estimated using SGE and GTE. Thus, we explore how eye gaze complexity may reflect the organisation of behaviour and individual experience in the context of driving. To test whether SGE and GTE correlate with years of driving experience, we collected a large dataset and analysed the dynamics of eye gaze in participants performing driving tasks in a simulator and on-road urban environments.

## 2. Materials and Methods

### 2.1. Participants

Participants of this study were over 18 years old and had a valid driving licence obtained at least one year prior to enrolling in one of our simulator- and on-road experiments. Only participants with sufficient quality of eye gaze recordings were included in the analyses (please see Section 2.4 for the criteria and eye gaze quality details): the final simulator sample consisted of 380 participants (56% male; aged between 20 and 73 years: Med = 45, M = 47, and SD = 11.54), and the final on-road sample contained 241 participants (56% male; aged between 19 and 74 years: Med = 44, M = 45, and SD = 11.87). The samples in the simulator and on-road experiments did not overlap. All participants confirmed they owned a car that they drive daily and specified that they primarily drive on urban roads. The participants reported driving experience ranging from 1 to 57 years (simulator: 1 to 57 years: Med = 20, M = 23, and SD = 12.72; on-road: 1 to 52 years: Med = 21, M = 22, and SD = 12.55). All participants reported they were healthy, without known neurological impairments or diagnoses, and not currently taking any psychoactive medication. All participants had normal-to-corrected vision and reported no colour blindness. In this study, driving experience was defined as years since obtaining a driving licence and typical urban driving frequency; no additional detailed driving history information (e.g., annual mileage, night driving, crash or near-miss history) was collected.

All experimental protocols and consent procedures were approved by the National Centre for Bioethics of the Scientific Psychology Centre of Yerevan State University (2 June 2023, No23/05/01). All participants signed an informed consent and were paid for taking part in this study. The study was conducted in accordance with the Declaration of Helsinki and all relevant regulations and guidelines.

### 2.2. Driving Simulation Experiments

The experiment was conducted in a fixed-base driving simulator developed using the BeamNG.py library (BeamNG.tech, version 0.23.5.1). Conventional in-vehicle equipment was included: a steering wheel, a driver’s seat, and pedals (acceleration and brakes, as in an automatic transmission). A computer with high processing capability (CPU: Intel Core i9-13900K, RAM: 64 GB, GPU: NVIDIA RTX 4090) was synchronised with the simulator to record participants’ steering activity and vehicle location along the x, y, and z axes. The simulator generated images on three LED monitors located in a 180° semicircle around the participant at a distance of approximately 1120 mm. Room temperature and lighting were controlled during the experiment (19–21 °C, 80–100 lux). The in-vehicle acoustic environment was simulated using standard BeamNG sound effects.

**Figure 1:**
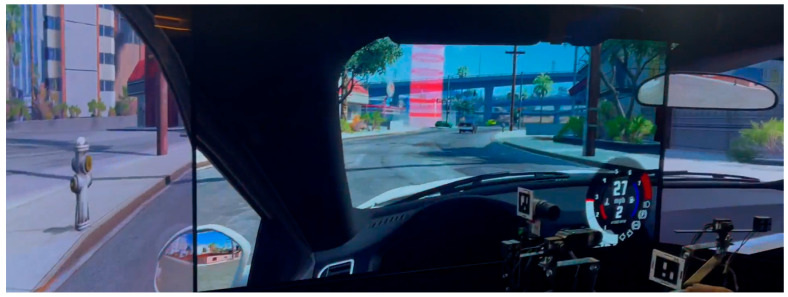
Simulated urban environment.

A standard city environment with traffic was simulated (Figure 1). Participants were instructed to drive along the route indicated by red markings on the road and to respect the speed limit and traffic rules. No feedback on driving performance or violations was provided. The set speed limit was 45 mph (72 km/h). In the rare event of accidental damage to the simulated vehicle, it was reinitiated from the last checkpoint so the participant could continue driving. This design choice was made to maintain a consistent effective exposure time for each participant. Reset events were rare in the present sample and therefore affected only a very small fraction of the data.

To minimise the impact of circadian rhythms, all experiments were performed between 8 a.m. and 6 p.m. Participants voluntarily chose a session that suited them best within the 2 h time slots available in the schedule. Prior to the experiments, participants were asked to ensure they had the usual amount of sleep the night before the experiment; avoid consuming food and drinks that contain caffeine and avoid taking any drowsiness-causing medications at least 8 h before the experiment; avoid consuming any alcohol at least 24 h before the experiment; not to smoke or engage in demanding physical activities at least 2 h before the experiment.

To confirm that the participants followed the instructions in preparation for this study, at the beginning of each experiment, participants were asked to complete a short questionnaire gathering information on their previous night’s sleep, caffeine and alcohol consumption, physical activity, smoking, and any medication taken. If participants violated the set preparatory conditions, they were excluded.

Participants were given the opportunity to familiarise themselves with the simulator by completing a 5 min practice driving task. In this work, we report only the results from the urban driving stage without additional tasks, which lasted 5 min. This time period was chosen because it is long enough to collect sufficient data for calculating eye gaze entropy measures while controlling for the effects of fatigue.

### 2.3. On-Road Driving Experiments

The on-road study was designed to be as close as possible to real-life driving. Real accident risks created higher driver motivation, which is not always possible to achieve in simulator studies. Variable environmental factors, such as traffic dynamics, weather conditions, unexpected road events, road closures, and repairs, were present. We used the same set of driving routes, but other factors and events were not under experimental control.

The participants drove a Mazda CX-5 with sensors and other equipment installed in the vehicle. The vehicle was equipped with a customised data acquisition system for time-synchronised recording of data from the steering wheel and pedals, an eye tracking system, a microphone, and a series of cameras for tracking various aspects of driver behaviour and the surrounding environment.

**Figure 2:**
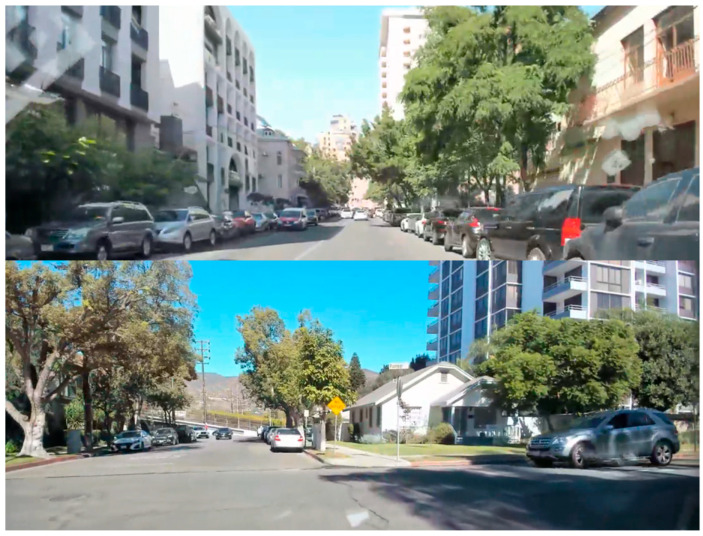
Real urban roads: screenshots from the experimental car’s dashcam in Yerevan, Armenia (**top**) and Northridge, CA, USA (**bottom**).

Participants were instructed to follow the directions of the navigation system, drive carefully given the weather conditions, and ensure that they obeyed the speed limit. The experiment was conducted on urban roads in Northridge (CA, USA) and Yerevan (Armenia) during the day (Figure 2). The participants were alone in the car while driving. It took participants 20–30 min to complete the urban route. There were two rest periods, before and after driving. During these periods, participants were asked to sit quietly in the car and listen to continuous waterfall sounds presented via the in-vehicle audio system. The waterfall soundscape served as a neutral, non-verbal auditory background to mask variable environmental noises and to help maintain a calm and consistent resting state across participants and locations. Data from these rest periods are part of the broader project dataset but are not included in the gaze-entropy analyses reported in this work.

As in the simulator experiment, participants had the same set of preparatory instructions regarding sleeping, eating, drinking, smoking, etc., and they answered questions prior to the experiment to confirm these instructions were not violated.

### 2.4. Eye Gaze Recording and Metric Calculations

In both types of experiments, eye gaze was recorded using a commercial eye gaze tracker, Smart Eye (https://smarteye.se/, accessed on 22 October 2025). Two cameras for gaze tracking were installed within a horizontal black bar attached to the bottom of the central screen, above the steering wheel. Smart Eye sampling rate was 60 Hz. Before each experiment, the eye tracking system was geometrically calibrated using the standard Smart Eye chessboard procedure to align the cameras and define the 3D tracking volume. Participants also completed a standard multi-point gaze calibration prior to driving in the simulator. Calibration was repeated when necessary if the experimenter observed clear misalignment between the estimated gaze position and visible screen or road features. According to the manufacturer’s specifications under recommended conditions, typical gaze accuracy is within approximately 0.5–1.0° of visual angle with comparable precision during stable fixations. Data was exported to custom-made Python (version 3.12.5) routines for subsequent analysis of saccades, fixations, and blinks. We used the internal quality parameters of the eye gaze tracking system to filter out invalid data. For each frame, the Smart Eye system outputs several data quality variables, including head position quality and gaze direction quality, each ranging from 0 (tracking failed) to 1 (optimal tracking) and reflecting the number of cameras contributing to the estimate and the strength of iris detection, respectively. Frames with low head position quality or gaze direction quality below a set threshold (<1.0), or flagged as invalid (e.g., tracking loss), were discarded. Only frames classified as valid were included.

To analyse the data, a sliding 10 s window with a 1 s step was employed. This approach is applied with a view to developing continuous driver monitoring, and it allows for effective control of eye gaze data quality during analyses. Only frames classified as valid contributed to each 10 s window, and only windows with a quality of raw gaze data higher than 50% were then selected for the analysis. This window approach is an efficient way to manage noise and artefacts in the gaze data when calculating metric values.

For each of these windows, a reference plane was constructed by averaging the head direction vector at a fixed distance over the entire window. The configuration of the plane was based on the equation of the plane through the normal vector to the plane and a point:

$$A\left(x-x_{0}\right)+B\left(y-y_{0}\right)+C\left(z-z_{0}\right)=0,$$

where $\left(x_{0},\;y_{0},\;z_{0}\right)$ is the point of the gaze plane and $\left(A,B,C\right)$ is the normal vector.

The normal vector was derived from the average head direction vector from the sliding window. The point of the gaze plane is a point plotted along the direction vector of the head from the position of the head to a given distance (in our case, the distance was 112 cm). Next, we determined intersections of the gaze direction vectors with the reference plane. To achieve this, we used Gaussian elimination to solve a system of linear equations in the following form:

$$\left[\begin{array}{ccc} gaze\_direction[1] & gaze\_direction[0] & 0\\ 0 & gaze\_direction[2] & -gaze\_direction[1]\\ A & B & C \end{array}\right]\left[\begin{array}{c} x\\ y\\ z \end{array}\right]=\left[\begin{array}{c} gaze\_direction[1]*gaze\_position[0]-gaze\_direction[0]*gaze\_position[1]\\ gaze\_direction[2]*gaze\_position[1]-gaze\_direction[1]*gaze\_position[2]\\ D \end{array}\right]$$

In this approach, the matrix does not have a solution using the Gaussian elimination method, only in two cases: when the line lies in the plane and when they are parallel. Based on the points of intersection of the eye direction vectors with the mobile projection plane, the main events of the eye movement are then calculated within each window following standard procedures [34,35]. At 60 Hz sampling, fixations were defined as periods during which the change in gaze direction between successive samples was <1° for at least 6 consecutive frames (≥100 ms), with an upper duration limit of 600 frames (10 s) to match the window length. Saccades were defined as periods with inter-sample gaze direction changes ≥1°, durations between 2 and 12 frames (≈30–200 ms), and total amplitude ≤60°, and were further filtered using a velocity-dependent threshold and an amplitude-to-peak-velocity ratio (AVR) ≤10 to reject implausible events. Blinks were identified from the eyelid-opening signal using derivative and duration thresholds (5–180 frames, ≈83–3000 ms). All parameters were kept constant across participants and conditions.

For each participant, we calculated the proportion of low-quality windows across the drive. Participants were retained in the analyses only if no more than 35% of their windows were low-quality. Among the included participants, the mean proportion of discarded low-quality windows was 12% (SD = 9%; range = 0–34%) in the simulator sample and 25% (SD = 6%; range = 11–34%) in the on-road sample.

Eye gaze entropy quantitatively assesses visual scanning during engagement in tasks involving high visuospatial demand. The entropy concepts used in application to eye gaze describe the amount of information required to generate a given sequence as a measure of overall uncertainty. To calculate SGE and GTE, fixation coordinates were discretised by organising them into spatial bins of 30 × 30 pixels for the generation of state spaces across the visual field with sufficient transition distributions. This binning approach follows previous work on gaze entropy in naturalistic driving tasks, where fixation coordinates were first discretised into pixel-based grids and, for fine-grained prediction analyses, entropy was computed using 30 × 30 pixel bins in short temporal windows [27]. The choice of a 30 × 30 grid reflects a compromise between spatial resolution and data sparsity. As noted in gaze-transition entropy work, increasing the number of grid cells or AOIs inflates the number of possible transitions and can lead to rows or cells with zero counts, which destabilizes entropy estimates and statistical comparisons [15,36]. The described data-driven sampling of the visual field is agnostic to specific semantic Areas of Interest (AOIs). We did not explicitly label or group bins into predefined driving-related regions such as roadway, mirrors, dashboard, etc. Accordingly, SGE and GTE are interpreted as measures of the dispersion and transition structure of gaze within the projected field of view, rather than as direct estimates of attention allocated to particular functional AOIs. Additionally, because fixation coordinates are obtained by intersecting 3D gaze vectors with a 2D reference plane at a fixed distance from the head, depth information in the underlying 3D driving environment is compressed, and the absolute SGE and GTE values are specific to this 2D projection.

SGE is a measure in which Shannon’s entropy equation is applied to the probability distribution of fixation coordinates to calculate the average level of uncertainty in the spatial distribution of a sequence of fixations generated in a given timeframe. Higher entropy or uncertainty, in this case, indicates a wider distribution of fixations across the visual field, suggesting greater dispersion of gaze. Stationery entropy is calculated as:

$$H_{s}\left(x\right)=-\sum_{i=1}^{N}p(i)\log_{2}p(i)$$

where $H_{s}\left(x\right)$ is the entropy value of a set represents the state spaces or the location (coordinates in a 2D plane) of each fixation contained in x, N is the total number of fixations within x, and p is the proportion of fixations landing on a given state space within x.

GTE builds on the stationary distribution of fixations to examine patterns in visual scanning. The conditional entropy equation (see below) is applied to Markov chain matrices of fixation transitions. An average measure of the predictability of visual scanning patterns is estimated. Higher entropy suggests a more random and unpredictable pattern of scanning behaviour. GTE was estimated by applying the conditional entropy equation to 1st order Markov transitions of fixations as follows:

$$H_{c}\left(x\right)=-\sum_{i=1}^{N}p(i)\left[\sum_{j=1}^{N}p(i|j)\log_{2}p(i|j)\right]$$

where p(i) is the stationary distribution of fixation locations, p(i|j) is the probability of transitioning to j given the current position of i, and i≠j denotes exclusion of transitions within the same state space from the inner summation.

Thus, SGE and GTE were calculated within rolling 10 s windows with a 1 s step, along with some other basic metrics: fixation number, fixation duration, and saccade amplitude. At a sampling rate of 60 Hz, a 10 s window contains up to 600 frames, which provides sufficient data to estimate both spatial and transition probability distributions within each window while still allowing temporal variation in gaze behaviour to be captured over time (e.g., see [18,21]). The 1 s step between windows yields overlapping segments and a smoother time series, reducing sensitivity to the arbitrary placement of non-overlapping windows.

In simulator experiments, windows in the initial 30 s of driving were excluded. The remaining window values were averaged for each participant, and the averaged values were correlated with driving experience and compared between groups.

In on-road experiments, as this was a more challenging task to drive an unfamiliar vehicle along an unfamiliar route, and there was no separate training time allocated, we considered the first 15 min of driving as such a training period and excluded it from analyses. To match the driving time to the simulator experiments, the following 5 min of driving were taken into analysis. All windows within that time period with acceptable quality were averaged for each participant, and the averaged values were correlated with driving experience and compared between groups. There was no way of controlling participant fatigue, which is much more likely to occur in the on-road setting, compared to the simulator task.

### 2.5. Statistical Analysis

Statistical analyses were performed using the open-source Python (version 3.12.5) SciPy library. T-test was used to compare between groups with large samples. Cohen’s (d) coefficient was used to evaluate the effect size. Mann–Whitney U test was used to compare between groups with small samples with distributions significantly deviating from the normal distribution. Common Language Effect Size (CL) was used to evaluate effect size. Spearman’s rank correlation coefficient was computed for the analysis of relationships between variables. We used an alpha level of 0.05 for all statistical tests.

## 3. Results

**Figure 3:**
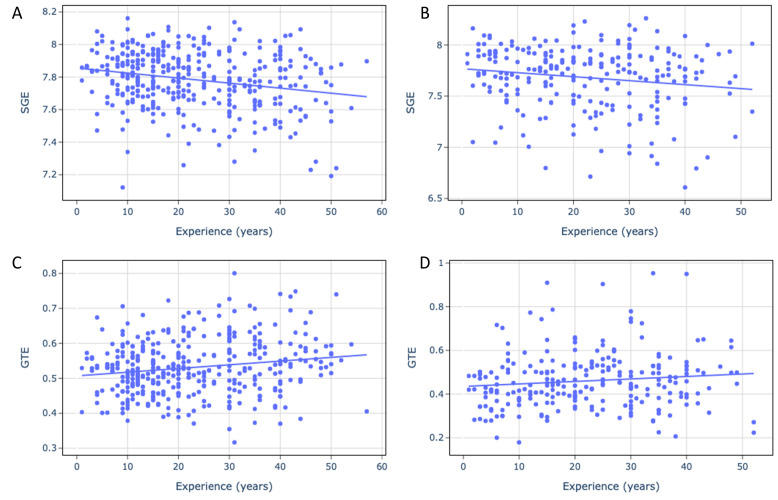
Scattergrams for driving experience and SGE (**A**,**C**), GTE (**B**,**D**) values in simulation (**A**,**B**), and on-road (**C**,**D**) experiments. Each dot represents one observation (one participant). The solid line indicates the fitted trend (the least-squares linear regression) to aid visual interpretation of the association; correlation was assessed using Spearman’s rank correlation.

Significant correlations were found between eye gaze entropy measures and driving experience in both simulator and on-road environments (Table 1, Figure 3). Years of driving experience consistently correlate with decreases in SGE and saccade amplitude, along with increases in GTE. No significant correlations were found for fixation duration. While saccade amplitude is also shown to be lower in older drivers, only years of driving experience significantly correlated with the gaze entropy measures.

Additionally, to examine whether the relationship between driving experience and gaze entropy was consistent across on-road locations, we repeated the correlation analyses separately for Yerevan and Northridge. In Yerevan, SGE showed a small negative correlation with experience (r = −0.14, *p* = 0.09), and GTE showed a positive correlation (r = 0.17, *p* = 0.04). In Northridge, the corresponding correlations were in the same direction (SGE: r = −0.13, *p* = 0.20; GTE: r = 0.16, *p* = 0.10). Thus, splitting the data by site attenuates the statistical strength of the effects, likely due to reduced sample size, but the overall pattern is preserved in both locations: lower SGE and higher GTE with greater driving experience.

No significant difference was observed in SGE and GTE measures between male and female participants in the simulator (SGE: *t*-test = −0.961, *p* = 0.337; GTE: *t*-test = 0.582, *p* = 0.561) or on-road experiments (SGE: *t*-test = 0.972, *p* = 0.332; GTE: *t*-test = −0.931, *p* = 0.353).

Additionally, we compared entropy measures between two groups of participants that were balanced with respect to their age but had different driving experience: Group 1 ≤ 7 years and Group 2 ≥ 13 years. As our sample contained fewer inexperienced drivers (Group 1 comprised 29 participants in the simulation experiment and 37 participants in the on-road experiment, while Group 2 contained 278 participants in the simulation and 177 in the on-road study), we balanced the samples by randomly selecting data from participants with the same gender and age from the bigger group to match the smaller sample. The final compared samples included: 29 participants in Group 1 and 25 participants in Group 2 for simulation; and 37 participants in Group 1 and 20 participants in Group 2 for the on-road study. As shown in Table 2, SGE values were lower in more experienced drivers, while no difference was observed in GTE. There was no difference in age distributions between the groups in simulation experiments (U = 310.5, *p* = 0.129). In on-road experiments, there was no difference in age distributions between the groups (U = 446, *p* = 0.117), but SGE was lower, and GTE was higher in more experienced drivers. In the more experienced group, fixation duration was higher, and fixation number was lower, but only in the on-road condition. 

**Figure 4:**
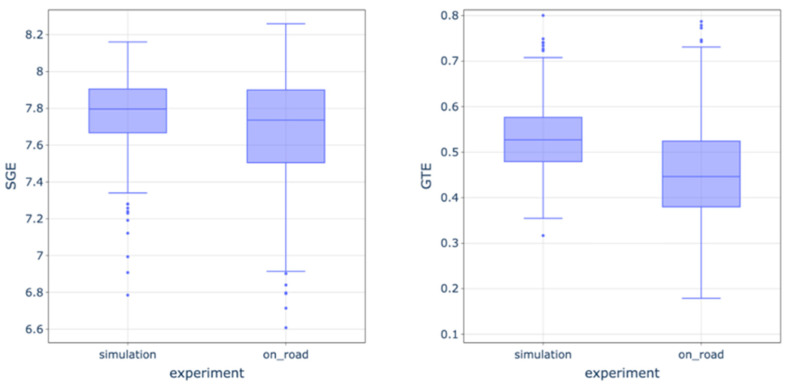
SGE and GTE values in simulation and on-road experiments. Boxes show the inter-quartile range (IQR, 25th–75th percentiles) with the median as the central line; whiskers extend to values within 1.5× IQR. Dots denote outliers beyond the whiskers.

Finally, we compared the gaze entropy measures between groups in the simulator and on-road experiments. We found that SGE (*t*-test = −3.156, *p* = 0.002) and GTE (*t*-test = −8.210, *p* < 0.001) values were lower during on-road driving (Figure 4 and Table 3). As can be seen from Table 3, participants made more fixations with shorter fixation durations in the on-road experiments in comparison to the simulator, and their saccade amplitude was higher.

## 4. Discussion

In this study, we explored eye gaze entropy measures in the context of driving. Our results demonstrate that SGE and GTE during driving correlate with driving experience: unpredictability of visual scanning, as measured by GTE, is higher, while the overall gaze dispersion estimated by SGE is lower, in more experienced drivers of both genders. This effect is observed in the simulator as well as in real on-road driving, although entropy values obtained in naturalistic on-road conditions were generally lower.

These results are consistent with the idea that as drivers gain experience, they may learn to allocate attention more selectively to task-relevant parts of the visual scene, leaving other areas relatively unattended, which is reflected in lower overall gaze dispersion. At the same time, the results suggest that the transition pattern may become more complex in more experienced drivers, meaning that each subsequent fixation is less predictable. This is an indication of less stereotyped gaze behaviour, e.g., experienced drivers may not check some parts of the visual scene regularly, and their gaze behaviour can be less determined by repeated spatial patterns. All these results are in line with the literature on experience-related gaze dynamics [22], including that observed in driving tasks [9,37].

In our work, gaze entropy was computed on a grid of projected gaze locations without explicit mapping to semantically meaningful driving AOIs (e.g., roadway, mirrors, dashboard, etc.). As a result, SGE and GTE index the statistical dispersion and transition structure of gaze rather than validated attentional allocation to specific task-relevant objects or regions. Entropy values are also influenced by the geometry of the visual scene and by head-pose variability, because these factors shape the distribution of projected gaze samples across the reference plane. Importantly, all participants in our study were exposed to the same scene geometry and recorded with the same head-tracking and projection pipeline, which reduces the likelihood that geometry or head-pose variability alone accounts for the observed group differences. Nonetheless, changes in SGE or GTE cannot be uniquely attributed to improved or degraded visual scanning efficiency in the traditional AOI-based sense. Instead, we interpret these metrics as potential systems-level descriptors of how gaze sampling is organised within a given environment. The systematic differences observed between experience groups suggest that gaze behaviour changes with experience. Future work should be implemented to combine entropy-based measures with AOI or scene semantics analyses to more directly quantify task-relevant attentional allocation.

Importantly, we show that although age and experience are highly correlated variables, the eye gaze entropy dynamics is more strongly related to driving experience and less so to age. Indeed, experience forms with age, and separating these two factors is a special task, which we attempted in our additional analysis where we matched a small subgroup of individuals within the sample on gender and age but with different driving experience. Our results support the hypothesis that there are experience-related changes in eye gaze dynamics during driving.

Our results on the correlation between eye gaze entropy measures and driving experience are in line with the system-evolutionary theory [30,31], which explains how the neural and physiological organisation of behaviour represents the history of its development. Individual development can be considered as the process of adaptation and increasing differentiation in individual–environment interactions. Earlier-formed elements of experience underlie the formation of new elements, which integrate into the structure of individual experience, contributing to growth in its complexity. Our previous work has demonstrated how newly formed and more advanced skills are associated with increased heart rate complexity [20]. This work shows that the dynamics of eye gaze patterns can also be a valuable indicator of experience-related changes in behavioural organisation.

It is worth noting that, using the same equipment and methods, gaze entropy values were higher in the simulator than in real on-road driving. In fact, all the metrics differed significantly between the simulator and on-road experiments: drivers made more fixations, with higher saccade amplitudes and shorter fixation durations on the road. Unlike some previous research [38,39], we did not specifically match any visual parameters of the simulator environment to those of the real driving route, because these were two separate studies. Therefore, we believe that these differences are primarily due to properties of the visual scene. Another factor is more focused attention on the road. Drivers usually concentrate on real roads more intensely; their arousal levels are higher, and they are more engaged in the driving task because it entails greater risks than simulator driving. Moreover, we believe drivers may have used side and rear-view mirrors more frequently on-road, because there was less need to use mirrors in our simulation setup. Finally, simulator driving is a novel task for most people: although they drive on-road in various urban environments daily, they do not often drive in a simulator, and for the majority of our sample this was a completely novel activity, which entailed learning. Research shows that the complexity of eye gaze and other physiological signals, such as heart rate variability [20], decreases at the beginning of learning [25].

Previous research comparing eye gaze in simulated and on-road driving shows that, while there is no significant difference in head movements [38] or the distance to fixations on target hazard stimuli [39], drivers have longer fixations [38,39] and higher saccade amplitudes in a simulator, which correspond to longer distances between successive fixations [38]. This is in line with our results on fixation duration. However, we observed the opposite effect for saccade amplitude, which was higher in real driving. This may be due to the specifics of the simulation setup and task. In our study, participants were instructed to follow a route highlighted on the simulator screen; therefore, participants tended to keep their gaze in the highlighted direction, which resulted in smaller saccade amplitudes compared to on-road driving, where the route was displayed on a navigation screen located in the vehicle cabin next to the driver. In the study by Robbins et al. [38], which found greater saccade amplitude in a simulator, the task was to drive according to verbal driving instructions in both the simulator and on-road environments.

Generally, a simulator environment has a number of differences from real on-road driving [38,39]. In particular, the visual scene is projected onto 2D screens to which the eyes are directed. In real driving, the 3D world has an additional depth parameter that is not considered, because all eye gaze metrics are calculated based on projections of the eye gaze vector onto a flat reference plane. In the simulator, the distance to this reference plane often corresponds to the distance to the simulator screens. On-road drivers tend to look at objects at greater distances. Generally, elements of the visual scene in real driving are located further away than in the simulator environment. Thus, the simulator’s 2D screens and the real-world 3D roadway environment differ in their visual-scene geometry, available depth cues, and peripheral structure. Such differences in visual-scene geometry are likely to affect gaze dispersion and transition patterns, and thus entropy. For these reasons, we cannot interpret quantitative contrasts in entropy between the simulator and on-road conditions directly. However, our current results may be used as a reference for generating hypotheses for future research in this direction.

The simulator route, on-road route, and on-road vehicle were unfamiliar to all participants prior to the study. This was intended to minimise the influence of route-specific expectations or prior experience. At the same time, this may have increased exploratory gaze and consequently affected the absolute level of gaze entropy. These factors likely contribute to the observed differences in entropy between environments. Accordingly, we interpret between-environment differences with caution and emphasise the within-environment associations between entropy and individual experience.

Thus, differences in eye gaze metrics between the simulator and on-road driving in our study could be accounted for by multiple factors, including the specifics of the visual scene, travelled routes and road environments, participants’ overall arousal and attention levels, and simulator task novelty, among other factors. Further, more controlled studies are needed to determine which simulator-specific aspects of eye movements should be considered when developing solutions for on-road use. However, most experience-related effects observed in the simulator in our study were also present in the dynamics of these metrics on the road. This suggests that eye tracking data obtained in a simulator can be used in the modelling of real road driving, with appropriate calibration.

Overall, the results of this study are informative for the development of solutions for driver state detection and other human–machine interactions. This is particularly relevant for fast-evolving artificial intelligence technologies. Eye gaze metrics, including entropy measures, can be extracted using driver monitoring system cameras that are being installed in an increasing number of vehicles. Eye gaze entropy measures have demonstrated their applicability in differentiating levels of cognitive workload [9,40], drowsiness [27], and psychoactive substance intake [15]. Incorporating driving experience into these algorithms could improve their accuracy and reliability. Additionally, we demonstrate that, although there are considerable differences between the eye gaze metrics collected in simulator studies compared to on-road driving, the dynamics of these metrics are largely comparable. Thus, our results reinforce previous research suggesting that eye gaze data obtained in simulators can be used to develop applications for on-road conditions, although calibration may be required.

### Limitations

The main limitation of our study is its independent-samples design. First, our analyses of experience were cross-sectional: we correlated measures of eye gaze complexity with years of driving experience across participants, but we cannot directly observe changes in eye gaze complexity as driving experience is gained. Ideally, our results need to be verified in a within-subjects study in which the same participants perform a driving task multiple times, for example, when they first obtain their driving licence and after one or more follow-up periods, to test whether the trends in the dynamics of eye gaze complexity shown in our study would hold. Secondly, the simulator and on-road datasets were obtained from different participant samples, rather than from the same drivers performing both tasks. This means that differences in gaze entropy between environments may partly reflect unmeasured group-level differences as well as properties of the driving context. A within-subjects design, or at least a subset of participants completing both simulator and on-road conditions with more closely matched task parameters, would allow stronger conclusions about the comparability and generalizability of entropy-based gaze metrics across driving environments. Organising such a longitudinal and within-subjects study is very challenging in practice; therefore, in the present work, we performed between-subjects analyses in relatively large samples to test for trends in experience-related dynamics of eye gaze complexity within each environment.

A further limitation of the present work concerns familiarity with the driving contexts. In both the simulator and the on-road experiments, the routes and the on-road vehicle were unfamiliar to participants. This design reduces potential confounds related to route-specific memory or prior expectations, but it may also increase information demands and promote more exploratory gaze behaviour, thereby influencing the absolute values of gaze entropy.

In addition, differences in visual-scene geometry are likely to affect gaze dispersion and transition patterns, and thus entropy. For these reasons, we are unable to interpret quantitative contrasts in entropy between the simulator and on-road conditions directly. Our primary conclusions focus instead on the sensitivity of entropy measures to individual experience within each environment, rather than on numerical comparisons of entropy across environments. Future research is needed to explore eye gaze specificity between the simulator and real on-road environments.

Another limitation is that we only analysed the gaze entropy measures during driving in urban environments, so we cannot draw conclusions about their dynamics in other road types, such as highways or rural roads. We hope to analyse experience-related eye gaze dynamics in other road environments in future studies.

Finally, we believe that other entropy measures could be useful for exploring different aspects of eye gaze complexity. We used two well-established measures, but there is research proposing new methodologies, such as quantifying eye gaze predictability by estimating active information storage, which accounts for temporal dependencies across multiple fixations [41].

## 5. Conclusions

The results of our work demonstrate that eye gaze entropy measures in the context of driving correlate with driving experience. Our findings suggest that entropy-based measures of gaze dispersion and transitions can capture systematic differences in how drivers sample the visual environment as a function of experience. In more experienced drivers, visual scanning, as measured by GTE, is less predictable and accompanied by lower overall gaze dispersion quantified by SGE, and this is observed within both simulated and on-road driving in urban environments. Overall, these findings are in line with the system-evolutionary theory, which describes how the organisation of behaviour reflects the history of individual development and how it corresponds to the growing adaptation and complexity of individual–environment interactions. Experience-related dynamics of eye gaze entropy can potentially be a useful factor in the development of practical applications, such as driver monitoring systems.

## Figures and Tables

**Table 1:** Spearman correlation (r) coefficients of the entropy measures with experience and age.

Eye Gaze Metric	Simulator		On-Road	
	Experience	Age	Experience	Age
SGE	r = −0.20, *p* < 0.001 *	r = −0.08, *p* = 0.103	r = −0.15, *p* = 0.024 *	r = −0.11, *p* = 0.106
GTE	r = 0.16, *p* = 0.002 *	r = 0.09, *p* = 0.059	r = 0.13, *p* = 0.037 *	r = 0.07, *p* = 0.315
Fixation number	r = 0.061, *p* = 0.128	r = 0.115, *p* = 0.004 *	r = 0.035, *p* = 0.537	r = 0.051, *p* = 0.378
Fixation duration	r = 0.08, *p* = 0.052	r = 0.025, *p* = 0.538	r = −0.034, *p* = 0.552	r = 0.14, *p* = 0.262
Saccade amplitude	r = −0.14, *p* = 0.001 *	r = −0.16, *p* = 0.001 *	r = −0.18, *p* = 0.001 *	r = −0.17, *p* = 0.002 *

* *p* < 0.05.

**Table 2:** Comparison of eye gaze metrics between two groups with different driving experience: Group 1 < 7 years and Group 2 > 13 years.

Eye Gaze Metrics	Simulation			On-Road		
	Mann-Witney Test	Median (Quartiles)		Mann-Witney Test	Median (Quartiles)	
		Group 1 (<7 Years)	Group 2 (>13 Years)		Group 1 (<7 Years)	Group 2 (>13 Years)
SGE	U = 583, *p* = 0.005 *, CL = 0.72	7.85 (7.77–7.94)	7.71 (7.62–7.80)	U = 785, *p* = 0.009 *, CL = 0.68	7.85 (7.69–7.93)	7.75 (7.43–7.81)
GTE	U = 352, *p* = 0.393, CL = 0.43	0.53 (0.46–0.55)	0.52 (0.49–0.61)	U = 365, *p* = 0.010 *, CL = 0.32	0.41 (0.32–0.47)	0.44 (0.41–0.55)
Fixation number	U = 2259, *p* = 0.090, CL = 0.59	1.66 (1.46–1.94)	1.43 (1.28–2.02)	U = 1474, *p* = 0.009 *, CL = 0.65	2.28 (1.91–2.67)	1.93 (1.55–2.26)
Fixation duration	U = 1666, *p* = 0.204, CL = 0.43	600.64 (512.85–675.57)	681.16 (438.31–812.08)	U = 771, *p* = 0.008 *, CL = 0.34	385.97 (311.02–500.62)	485.31 (398.91–858.83)
Saccade amplitude	U = 2181, *p* = 0.192, CL = 0.57	33.15 (29.27–36.36)	30.97 (25.87–34.83)	U = 1385, *p* = 0.055, CL = 0.61	38.01 (32.46–43.56)	35.46 (31.19–40.59)

* *p* < 0.05.

**Table 3:** Comparison of eye gaze metrics between simulator and on-road experiments.

Eye Gaze Metrics		Simulator		On-Road	
	*t*-Test	Mean	Std	Mean	Std
SGE	t = −3.16, *p* = 0.002 *, d = 0.26	7.76	0.36	7.66	0.33
GTE	t = −8.21, *p* < 0.001 *, d = 0.68	0.52	0.08	0.46	0.12
Fixation number	t = −214.59, *p* < 0.001 *, d = 0.68	1.75	0.74	2.31	0.91
Fixation duration	t = 89.37, *p* < 0.001 *, d = 0.28	648.67	597.47	448.07	812.74
Saccade amplitude	t = −100.72, *p* < 0.001 *, d = 0.32	31.63	12.97	36.24	15.85

* *p* < 0.05.

## Data Availability

The datasets presented in this article are not readily available because of ethical and legal restrictions. Requests to access the datasets should be directed to the corresponding author.

## Abbreviations

The following abbreviations are used in this manuscript:

GTE	Gaze Transition Entropy
SGE	Stationary Gaze Entropy
AOI	Area of Interest
